# Responsiveness of metallothionein and hemocyanin genes to cadmium and copper exposure in the garden snail *Cornu aspersum*

**DOI:** 10.1002/jez.2425

**Published:** 2020-11-03

**Authors:** Veronika Pedrini‐Martha, Raimund Schnegg, Gabriela Giannina Schäfer, Bernhard Lieb, Willi Salvenmoser, Reinhard Dallinger

**Affiliations:** ^1^ Department of Zoology and Center of Molecular Biosciences Innsbruck University of Innsbruck Innsbruck Austria; ^2^ Institute of Molecular Physiology, Johannes Gutenberg‐University of Mainz Mainz Germany

**Keywords:** bioaccumulation, Gastropoda, metal metabolism, respiration, stress

## Abstract

Terrestrial gastropods express metal‐selective metallothioneins (MTs) by which they handle metal ions such as Zn^2+^, Cd^2+^, and Cu^+^/Cu^2+^ through separate metabolic pathways. At the same time, they depend on the availability of sufficient amounts of Cu as an essential constituent of their respiratory protein, hemocyanin (Hc). It was, therefore, suggested that in snails Cu‐dependent MT and Hc pathways might be metabolically connected. In fact, the Cu‐specific snail MT (CuMT) is exclusively expressed in rhogocytes, a particular molluscan cell type present in the hemocoel and connective tissues. Snail rhogocytes are also the sites of Hc synthesis. In the present study, possible interactions between the metal‐regulatory and detoxifying activity of MTs and the Cu demand of Hc isoforms was explored in the edible snail *Cornu aspersum*, one of the most common European helicid land snails. This species possesses CdMT and CuMT isoforms involved in metal‐selective physiological tasks. In addition, *C. aspersum* expresses three different Hc isoforms (CaH ɑD, CaH ɑN, CaH β). We have examined the effect of Cd^2+^ and Cu^2+^ exposure on metal accumulation in the midgut gland and mantle of *C. aspersum*, testing the impact of these metals on transcriptional upregulation of *CdMT*, *CuMT*, and the three *Hc* genes in the two organs. We found that the *CuMT* and *CaH ɑD* genes exhibit an organ‐specific transcriptional upregulation in the midgut gland of Cu‐exposed snails. These results are discussed in view of possible interrelationships between the metal‐selective activity of snail MT isoforms and the synthesis and metabolism of Hc isoforms.

## Highlights

- Cadmium and copper are accumulated in the midgut gland and mantle of *Cornu aspersum*.
- Metallothionein (MT) genes show differential metal‐dependent upregulation patterns.
- Hemocyanin gene expression can be upregulated due to copper exposure.

## Graphical abstract

In the terrestrial snail *Cornu aspersum*, cadmium (Cd) and copper (Cu) are mainly accumulated in the midgut gland and mantle tissues, leading to transcriptional upregulation in the midgut gland of *Cd‐Metallothionein* after Cd exposure, and of *Cu‐Metallothionein* and a *hemocyanin* gene after Cu exposure.

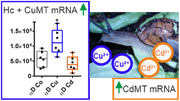

## 1 INTRODUCTION

Terrestrial gastropods possess sophisticated mechanisms for diverting essential and nonessential transition metal ions such as Zn^2+^, Cd^2+^, and Cu^+^/Cu^2+^ through separate metabolic pathways (Dallinger, 1993; Dvorak et al., 2019). A crucial role in this regulation is attributed to snail metallothioneins (MTs; Dallinger et al., 1997) which can specifically bind either Cd^2+^ and Zn^2+^ or Cu^+^ through activation of metal‐selective isoforms (Palacios et al., 2011). The Cu‐specific MT (CuMT) of terrestrial helicid snails, in particular, has been suggested to play a role in the synthesis of hemocyanin (Hc; Dallinger et al., 2005), the Cu‐binding respiratory protein of most snails and molluscs (Kato et al., 2018; Lieb et al., 2010). In fact, snail CuMT is exclusively expressed in rhogocytes (Chabicovsky et al., 2003), which represent a particular molluscan cell type that occurs in the primary body cavity, either freely floating in the hemocoel or embedded in connective tissues (Haszprunar, 1996; Stewart et al., 2014). Apart from CuMT, rhogocytes are also the sites of Hc synthesis, as demonstrated for several Hcs and Hc isoforms in different gastropod species (Albrecht et al., 2001; Martin et al., 2011; Sminia & Boer, 1973; Sminia & Vlugt‐Van Daalen, 1977). In the mantle of *Haliotis laevigata*, for example, two morphologically distinct rhogocyte populations were identified which probably represent two separate phases of Hc synthesis (Sairi et al., 2015).

However, the hypothesis of CuMT involvement in gastropod Hc synthesis is compromised by the fact that many species of Gastropoda do not possess Cu‐selective MTs (Dallinger et al., 2020), and some do not use Hc as a primary respiratory protein (Alyakrinskaya, 2002; Lieb et al., 2006). Yet, this does not preclude an interaction between CuMTs and Hc synthesis, in species which express both, Cu‐selective MTs and Hcs, in the same rhogocyte cells. Apart from Hc synthesis, gastropod rhogocytes have long been known to be involved in homeostasis and/or detoxification of trace metals (Kokkinopoulou et al., 2014, 2015; Marigómez et al., 2002; Simkiss & Mason, 1983). Because of the indispensable role of copper (Cu^+^/Cu^2+^) as a central component of Hc in most snails, the homeostatic regulation of this metal may be crucial. Metal balance analyses of snail tissues indicate that Cu is accumulated, among other organs, in the midgut gland and mantle (Berger & Dallinger, 1989), in which a particularly high density of rhogocytes can be detected (Sairi et al., 2015). Chromatographic fractionations show, moreover, that snail tissue Cu is mainly allocated to Hc and MT fractions (Berger et al., 1997; Dallinger, 1996), even upon exposure to nonessential trace elements like cadmium (Cd; Dallinger et al., 2004). Yet, Cd exposure can significantly impact on Cu metabolism and homeostasis in tissues of snails (Gnatyshyna et al., 2020; Nica et al., 2019) and other molluscs (Wu & Wang, 2010). In mammals, Cd interacts with important proteins and enzymes involved in Cu homeostasis, including MT, Cu‐chaperonins, and Cu‐transporters (Moulis, 2010). Overall, this may also apply to gastropods and particularly to snail species that express both Hc and a Cu‐selective MT isoform (Dallinger et al., 2005). Yet, information about possible interactions between metal accumulation (Cd^2+^, Cu^+^/Cu^2+^) and CuMT or Hc expression in snails is still poor.

The edible snail *Cornu aspersum* (the escargot) is one of the most common helicid land snails in Western and Central Europe. This species possesses CdMT and CuMT isoforms involved in metal‐selective physiological tasks such as Cd detoxification and homeostatic Cu regulation (Höckner et al., 2011; Palacios et al., 2011). In addition, *C. aspersum* expresses three different functional Hc isoforms (CaH ɑD, CaH ɑN, CaH β) (Schäfer et al., 2019). An involvement of CuMT in Hc synthesis of this species can therefore not be excluded. In the present study, we have examined the effect of Cd^2+^ and Cu^2+^ exposure on metal accumulation in the midgut gland and mantle of *C. aspersum*, along with the impact of these metals on transcriptional upregulation of *CdMT*, *CuMT*, and the three *Hc* genes in the two organs. Interestingly, the genes of CuMT and of CaH ɑD exhibit an organ‐specific expression pattern in the midgut gland of Cu‐exposed snails. These results are discussed in view of possible physiological interrelationships between the metal‐selective activity of snail MT isoforms and the synthesis and metabolism of Hc isoforms.

## 2 MATERIALS AND METHODS

### 2.1 Animal rearing and metal exposure

Specimens of *C. aspersum* (garden snail) were obtained from a commercial dealer (Wiener Schneckenmanufaktur e.U., Vienna, Austria). Before exposure, snails were kept for several weeks in groups on garden soil complemented with lime powder (CaCO_3_) under constant conditions (12:12 h photoperiod, 18°C ambient temperature) for acclimatization. They were fed with lettuce (*Lactuca sativa*) ad libitum. For exposure experiments, 30 snails were kept individually in octagonal plastic boxes (diameter 12 cm; height 6 cm) for 10 days under the same conditions (see also Pedrini‐Martha et al., 2016). Lettuce was metal‐enriched by soaking salad leaves for 1 h in Titrisol standard dilutions (Merck) of 2 mg/L CdCl_2_ (actual concentration: 130.12 ± 57.43 µg/g dry weight [d.w.] Cd) or 10 mg/L CuCl_2_ (actual concentration: 285.18 ± 77.28 µg/g d.w Cu), followed by 30 min of draining. Ten snails per treatment group were provided with metal‐enriched lettuce whereas controls were fed with untreated lettuce (Cd: 0.72 ± 0.31 µg/g d.w.; Cu: 9.68 ± 2.10 µg/g d.w.), every second day. Feeding behavior was monitored and documented before every new food supply. At the end of the exposure period, eight snails of each group (controls, Cd, Cu) were killed and dissected on an ice‐cooled aluminum plate, intermittently rinsed with RNase Away® Reagent (Ambion by Life Technologies, Thermo Fisher Scientific) and tissue samples of mantle edge and midgut gland were isolated. Snails which remained in a dormant condition during exposure and did not consume lettuce were excluded from further processing. Tissue aliquots for RNA isolation were stored in RNALater™ (Thermo Fisher Scientific) at −80°C until further processing. Tissue aliquots for metal analysis as well as leaves of *L. sativa* were immediately processed (see Section 2.2 and Pedrini‐Martha et al., 2016).

### 2.2 Metal measurement

All tissue (*n* = 8) and lettuce samples (*n* = 4) were oven‐dried at 60°C in 12 ml screw‐capped polyethylene rubes (Greiner). After d.w. determination, a 1:1 mixture of deionized water and nitric acid (65%) (Suprapur; Merck) was added and samples were heat‐digested at 70°C through several days until a clear solution was obtained. Samples were diluted with deionized water to a final volume of 11.5 ml. Cd and Cu concentrations of tissue and lettuce samples were analyzed by flame atomic absorption spectrophotometry (model 2380; Perkin Elmer). Samples with concentrations below the detection limits (e.g., Cd concentrations in mantle tissue) were remeasured by graphite furnace analysis (Z‐8200 Polarized Zeeman Atomic Absorption Spectrophotometer with SSC‐300 Auto Sampler; Hitachi). Instruments were calibrated with appropriate Cd and Cu solutions prepared from a Titrisol Cd or Cu standard solution (1000 mg/L; Merck). TORT‐2 Lobster Midgut gland (NRC) and Polish Virginia Tobacco leaves (INCT‐PVTL‐6; INCT) were used as standard reference materials for validation of metal measurement accuracy. Metal concentrations of standard reference materials were found to be within the accepted range (±10%) of certified metal values.

### 2.3 RNA extraction and complementary DNA (cDNA) synthesis

Total RNA extractions from six individuals of each exposure group were performed applying the RNeasy Plant Mini Kit (Qiagen) with On‐Column DNA digestion (Qiagen) according to the manufacturer's instruction with following modifications: For tissue homogenization, samples were homogenized with a Precellys 24 ball mill (Bertin Corp.) in 450 µl Buffer RLT+ 4.5 µl 2‐mercaptoethanol (Merck). Integrity of isolated RNA was verified by gel electrophoresis and quantified using the Quant‐iT™ RiboGreen® RNA assay kit (Invitrogen, Thermo Fisher Scientific). For cDNA synthesis 200 ng total RNA were applied using the SuperScirpt™ IV First‐Strand Synthesis System (Invitrogen, Thermo Fisher Scientific) following the manufacturer's instructions.

### 2.4 Mapping of RNAseq data against known cDNA sequences of Hcs and MTs of *C. aspersum*

Total RNA from midgut gland of two controls and two Cu‐exposed snails were sent to StarSeq for new generation sequencing (NGS) as described previously (Schäfer et al., 2019). Bioinformatic analyses of RNA sequences were performed using Geneious 9.1.8 (Kearse et al. 2012). Sequencing adapters of transcriptomic NGS data were removed and raw reads were quality‐trimmed. Trimmed reads of all four datasets were then mapped together to the three known Hc sequences from *C. aspersum* (CaH αD: MH485355, CaH αN: MH485356, CaH β: MH485357), to detect single nucleotide polymorphisms (SNPs). For each Hc isoform 20 sequence sections with a length of 45 nucleotides each that do not contain any SNPs were isolated as references for the quantitative analysis. The sequence sections were distributed over the complete coding regions but they were abundant at the same position with respect to the coding sequences of all *hemocyanin* genes to enhance comparability. For MT sequences, the CuMT (EF178297) and the CdMT (EF152281) were chosen as references for a prescreening to identify the allelic sequence variations for each individual to use them as references for the quantitative analysis. Trimmed reads of NGS datasets from different individuals were mapped separately from each other to these reference sequences of Hcs and MTs to obtain their relative quantity (given as transcripts per million) within the transcriptome of cupper fed individuals and those of the control group. Minimum coverage was set to 45 nucleotides and overlap identity to 100%.

### 2.5 Quantification of *MT* and *Hc* gene transcription via quantitative reverse transcription polymerase chain reaction (qRT‐PCR)

Gene‐specific primers of the *MT* (Höckner et al., 2011) and *Hc* genes for qRT‐PCR were designed using Primer Express 3.0 software (Applied Biosystems by Thermo Fisher Scientific; see Table 1). Primer dissociation curves were used to determine the optimal primer concentration (see Table 1). Calibration curves were generated by using cleaned PCR products for *Hc* genes (Qiagen PCR purification kit; Qiagen) or amplicon plasmids for *MT* genes. Cycle quantification (Cq) values were estimated as followed: CaH αN: *y* = −3.2929*x* + 32.692; CaH αD: *y* = −3.4732*x* + 35.011; CaH β: *y* = −3.5882*x* + 37.551; CdMT: *y* = −3.2775*x* + 37.382; CuMT: *y* = −3.3551*x* + 36.058. *MT* and *Hc* gene expression was quantified with the QuantStudio™ 3 (Applied Biosystems, Thermo Fisher Scientific) in a 10 µl approach applying the Power SYBR® Green PCR Master Mix (Applied Biosystems, Thermo Fisher Scientific). The respective transcripts were amplified using the following protocol: one initial denaturation step for 10 min at 95°C, 40 cycles denaturation for 15 s at 95°C and annealing/extension for 1 min at 60°C.

**Table 1:** Characterization of gene‐specific primers used for quantitative reverse transcription polymerase chain reaction

Primer	Sequence 5ʹ–3ʹ	Length (bp)	Conc. (nm)	Primer efficiency	Amplicon length (bp)
CdMT S	GCCGCCTGTAAGACTTGCA	19	900	101.89	56
CdMT AS	CACGCCTTGCCACACTTG	18	900		
CuMT S	AACAGCAACCCTTGCAACTGT	21	900	98.63	73
CuMT AS	CGAGCACTGCATTGATCACAA	21	900		
CaH αD S	CCCTGTCAGCAAGGACAATACC	22	900	94.05	62
CaH αD AS	CAATGCGGGTGCCTTTCTT	19	900		
CaH αN S	GCCCTGGTCCAATGAGATTCT	21	900	101.23	60
CaH αN AS	CCAGCTTGTCGGACTGCAT	19	900		
CaH β S	ATCCCAATTGGTGCTGAGAAA	21	900	89.97	60
CaH β AS	CGTGTCCTTGGGCACAATG	19	900		

*Note*: Sequences, lengths and primer concentrations are listed. Values for primer efficiency and amplicon length are also reported.

### 2.6 Statistical analysis

For statistical analysis and graphical drawings, the software package GraphPad Prism (Version 6.01; GraphPad Software Inc.) was used. Significant outliers were removed from the data set (Grubb's test, *p* < .05). Data were tested for normality (Shapiro Wilks test). For normal‐distributed data, one‐way analysis of variance statistics were applied. For intergroup comparison of all treatments, a Holm‐Sidak's multiple comparison test was performed. For comparison of control values with those of Cu‐ and Cd‐treated snails within a respective data set (e.g., *CdMT* gene expression of untreated and metal‐exposed snails in the midgut gland), a Dunnett's multiple comparison test was performed. For non‐normal‐distributed data, a Kruskal–Wallis test and the posthoc test Dunn's multiple comparison were applied. Significance level was set at *p* < .05.

## 3 RESULTS

### 3.1 Tissue‐specific accumulation of Cd and Cu by *C. aspersum*

**Figure 1:**
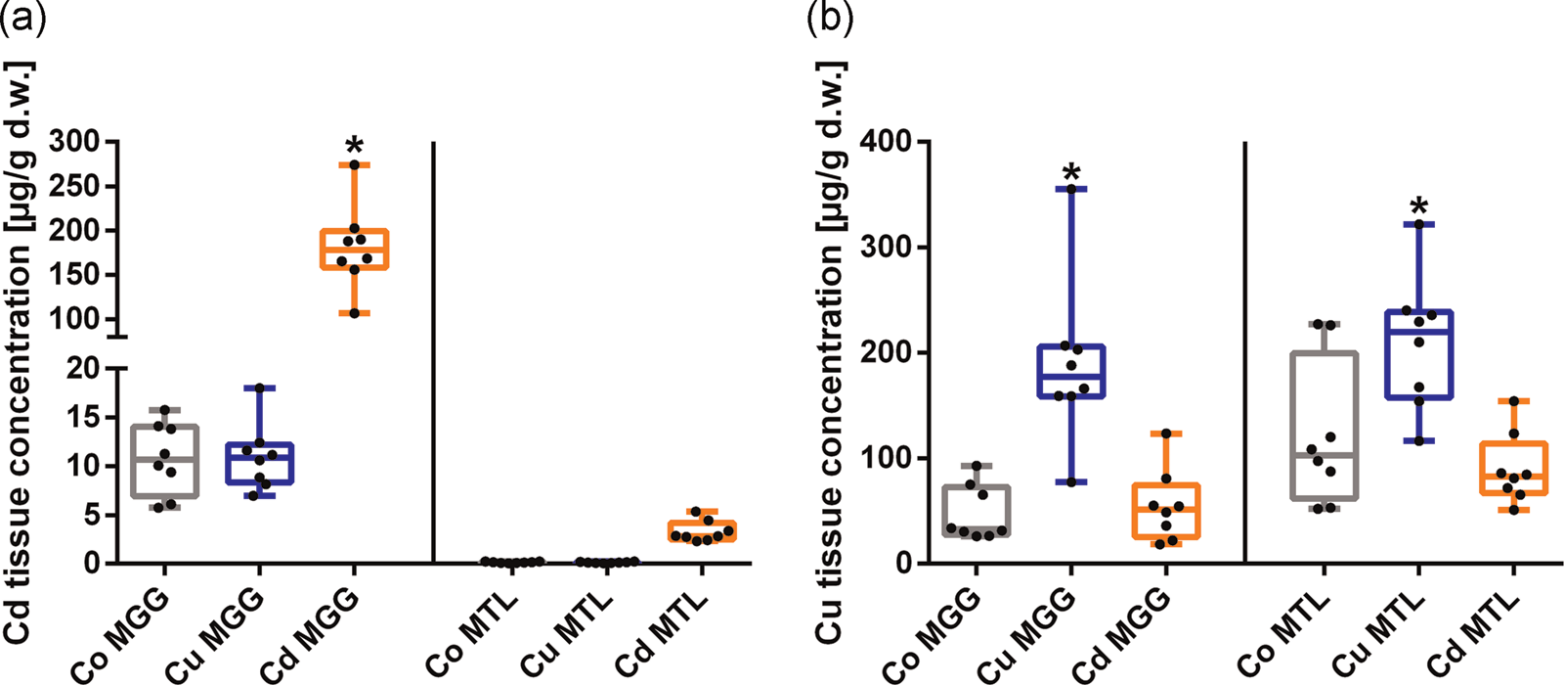
Metal accumulation in the midgut gland and mantle in *Cornu aspersum*. Whiskar box plots for Cd (a) and Cu (b) concentrations in the MGG and MTL (*n* = 8 each) of controls (Co; gray boxes), Cu‐exposed (blue boxes) and Cd‐treated snails (orange boxes) are shown. The respective boxes extend from the 25th to the 75th percentile, with square lines showing the medians. Single values of each group are represented by single black dots. *Indicate significance (*p* < .05) compared to control values (Holm‐Sidak's multiple comparison). MGG, midgut gland; MTL, mantle tissue [Color figure can be viewed at wileyonlinelibrary.com]

Adult snails of *C. aspersum* accumulated Cd and Cu in a tissue‐specific manner (Figure 1). Cd concentrations in the midgut gland tissue of exposed animals (181.46 ± 47.47 µg/g d.w.) were significantly higher compared to unexposed individuals (10.78 ± 3.68 µg/g d.w; Figure 1a and Table 2). Generally, Cd tissue concentrations in the mantle edge were significantly lower than midgut gland values, even after Cd exposure (Figure 1a and Table 2).

**Table 2:** Accumulation values for Cd and Cu in the midgut gland and mantle of *Cornu aspersum*

Tissue	Group	Cd (µg/g)	SD (%)	BAF	Cu (µg/g)	SD (%)	BAF
Midgut gland	Controls	10.78	34		47.83	55	
	Cu‐exp.	10.98	31	1	189.32	41	4
	Cd‐exp.	181.46	26	17	54.95	62	1
Mantle	Controls	0.14	47		121.51	57	
	Cu‐exp.	0.13	48	1	209.37	30	2
	Cd‐exp.	3.30	32	23	89.76	37	1

*Note*: Mean Cd and Cu tissue concentrations (µg/g d.w.) (*n* = 8) for controls and metal‐exposed snails are shown. For comparison of the variability of single values within each group, the respective standard deviation values are expressed in percent (*SD* [%]). In addition, the BAF reflects the metal accumulation in soft tissues of metal‐treated snails referred to controls.

Abbreviation: BAF, bioaccumulation factor.

In untreated snails, Cu concentrations in the mantle edge were considerably higher compared to those in the midgut gland (Figure 1b and Table 2). However, after Cu exposure, Cu concentrations of both tissue types were in an equal range (Table 2). Even though Cu was accumulated in both tissues types after Cu exposure, Cd showed a clearly higher bioaccumulation capacity than Cu, with bioaccumulation factor values of 17 and 23 in the midgut gland and mantle of Cd‐exposed snails, respectively (Figure 1b and Table 2). Cu tissue concentrations in Cd‐exposed animals and Cd tissue levels in Cu‐treated snails did not change in comparison to the respective concentration values of control individuals (Figure 1 and Table 2). Data also revealed that Cu tissue concentrations were more variable in the midgut gland compared to the respective Cd values (Table 2).

### 3.2 Basal and metal‐dependent *MT* gene expression in *C. aspersum*

**Figure 2:**
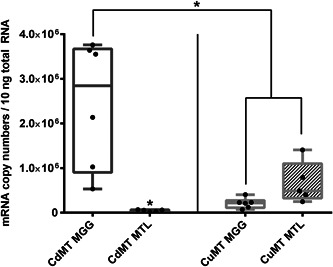
Basal expression of *MT* genes in the midgut gland and mantle of unexposed individuals of *Cornu aspersum*. Whiskar box plots show *CdMT* (left hand side) and *CuMT* (right hand side) gene transcription of untreated snails in MGG (*n* = 6) and MTL (*n* = 5). The respective boxes extend from the 25th to the 75th percentile, with square lines representing the medians. Single values are displayed as single black dots. For statistical analysis a Dunn's multiple comparison test was applied (see Section 2.6). *Indicate significance (*p* < .05). MGG, midgut gland; MTL, mantle tissue

The basal transcription of both *MT* genes showed an isoform‐ and tissue‐specific expression pattern in untreated snails (Figure 2).

**Figure 3:**
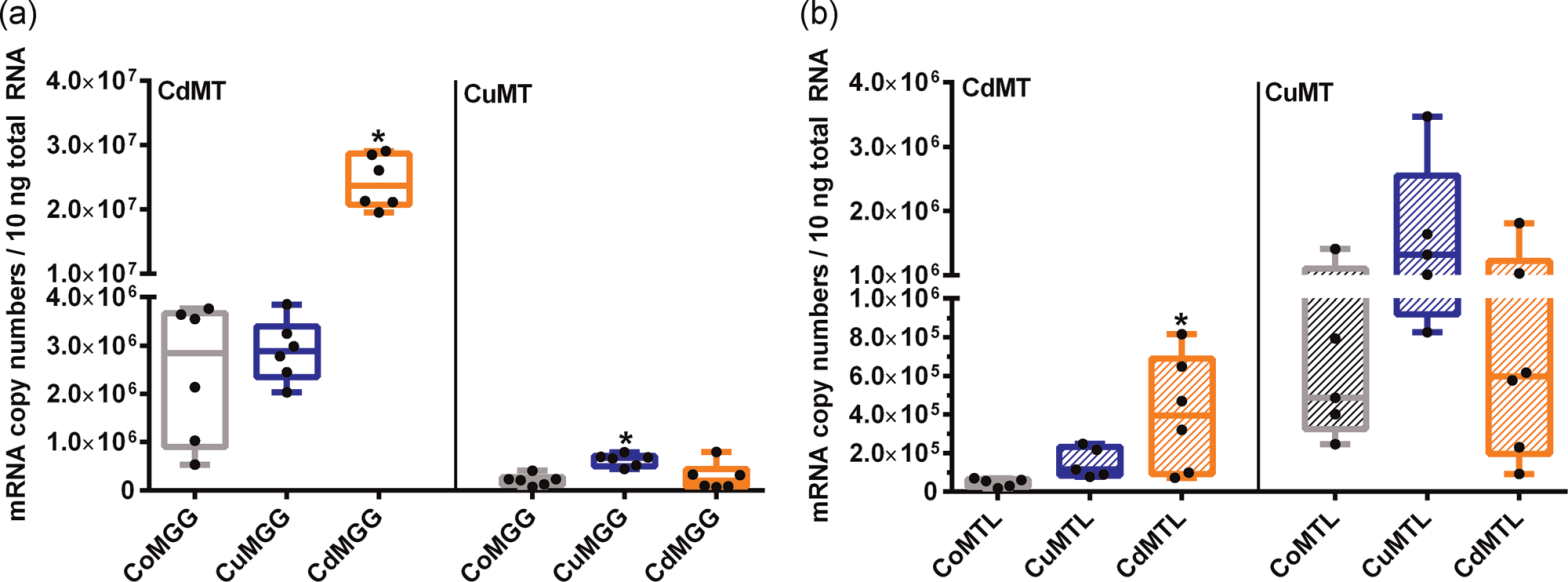
Metal‐dependent expression of *MT* genes in the midgut gland (a) and mantle (b) of *Cornu aspersum*. Whiskar box plots show quantification of *CdMT* and *CuMT* gene transcription in controls (Co) (gray boxes), Cu‐exposed (blue boxes) and Cd‐treated snails (orange boxes) (*n* = 5–6 per treatment group). The respective boxes extend from the 25th to the 75th percentile, with square lines showing the medians. Single values of each group are reported as single black dots. To analyze the impact of metal exposure on *CdMT* and *CuMT* gene expression the two tissue types were compared separately. For *CdMT* gene expression in the mantle and in the midgut gland, and for *CuMT* gene expression in the mantle, a Dunn's multiple comparison test was applied. For *CuMT* gene expression in the midgut gland, a Dunnett's multiple comparison test was applied (see Section 2.6). *Indicate significance (*p* < .05) within the respective data set [Color figure can be viewed at wileyonlinelibrary.com]

Whereas the *CdMT* gene was markedly higher expressed in the midgut gland than in the mantle (46‐fold), the *CuMT* transcripts appeared to be more abundant in the mantle, even though no significance could be detected. In addition, the *CdMT* gene in the midgut gland was 10‐fold higher expressed than the *CuMT* gene, whereas the *CuMT* gene expression in the mantle seemed to be more pronounced in comparison to the *CdMT* isoform (Figure 2). Upon 10 days of Cd exposure, *CdMT* gene expression was significantly upregulated by a 12‐fold increase in the midgut gland and an eightfold induction in the mantle tissue, when compared to controls (Figure 3).

In contrast, after Cu exposure the *CuMT* gene transcription in the midgut gland was upregulated only threefold (Figure 3a), whereas no significant increase could be detected in the mantle, even though the visual pattern may suggest a stimulating tendency of Cu exposure on *CuMT* gene transcription (Figure 3b). Overall, gene expression data suggested that only exposure to the cognate metal that is bound by the expressed metal‐selective MT isoforms can stimulate their respective mRNA transcription. In other words, only Cd exposure had a significant impact on *CdMT* gene expression, while Cu exposure lead to a significant increase of only *CuMT* gene transcription (Figure 3).

### 3.3 Basal and metal‐dependent *hemocyanin* gene expression in *C. aspersum*

**Figure 4:**
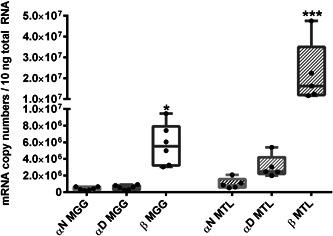
Basal expression of Hc genes in the midgut gland and mantle of unexposed individuals of *Cornu aspersum*. Whiskar box plots show the gene transcription of the three *Hc* genes *CaH αN*, *αD*, and *β* in the MGG (*n* = 6) (empty bars) and MTL (*n* = 5) (hatched bars) of untreated snails. The respective boxes extend from the 25th to the 75th percentile, with square lines showing the medians. Single values of each group are represented by single black dots. Data were not normal distributed, so a Dunn's multiple comparison test was applied (see Section 2.6). *Indicate significance (*p* < .05), with *meaning a significant difference between *β* MGG and *αN* MGG, and ***indicating a significant difference between gene transcription of *β* MTL compared to *αN* MGG, *αD* MGG and *αN* MTL. MGG, midgut gland; MTL, mantle tissue

All three *Hc* genes, namely *CaH αD*, *αN*, and *β*, showed varying basal transcription rates in untreated snails. Whereas the genes of *CaH αD* and *αN* exhibited nearly identical basal transcription levels in the midgut gland and slightly different expression rates in the mantle, *CaH β* transcripts were most abundant in both tissue types. *CaH β* exhibits markedly higher basal gene transcription levels compared to *CaH αD* (with factors of 10 in the midgut gland, and of seven in the mantle) and *CaH αN* (with factors of 13 in the midgut gland, and of 21 in the mantle; Figure 4). Moreover, transcription levels of the *CaH β* gene showed the highest variability range of single values measured in both tissue types (Figure 4).

**Figure 5:**
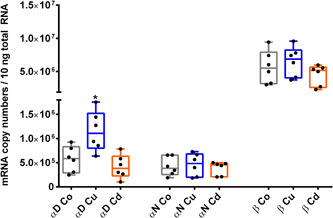
Metal‐dependent expression of *Hc* genes in the midgut gland of *Cornu aspersum*. Whiskar box plots show gene transcription patterns of the three *Hc* genes *CaH αD*, *αN*, and *β* in controls (Co) (gray boxes), Cu‐exposed (blue boxes) and Cd‐treated snails (orange boxes) (*n* = 5–6 per treatment group). The boxes extend from the 25th to the 75th percentile, with square lines showing the medians. Single values of each group are shown as single black dots. Data for *αD* and *αN* gene expression were analyzed by the Dunnett's multiple comparison test (data normal‐distributed), whereas metal‐dependent gene expression of the *Hc β* gene was analyzed with a Dunn's multiple comparison procedure (data not normal‐distributed) (see Section 2.6). *Indicate significance (*p* < .05) [Color figure can be viewed at wileyonlinelibrary.com]

**Figure 6:**
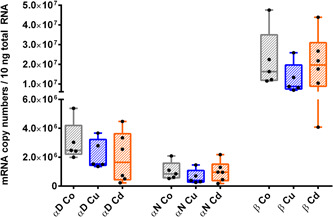
Metal‐dependent expression of *Hc* genes in the mantle of *Cornu aspersum*. Whiskar box plots show gene transcription patterns of the three *Hc* genes *CaH αD*, *αN*, and *ß* in controls (Co) (gray boxes), Cu‐exposed (blue boxes) and Cd‐treated snails (orange boxes) (*n* = 5–6 per treatment group). The boxes extend from the 25th to the 75th percentile, with square lines showing the medians. Single values of each group are shown as single black dots. All data of the respective *Hc* genes were analyzed using the Dunnett's multiple comparison test (see Section 2.6). *Indicate significance (*p* < .05) [Color figure can be viewed at wileyonlinelibrary.com]

A tissue‐specific view of metal‐exposed snails unveils that a significant transcriptional upregulation was observed for the *CaH αD* gene expression after Cu exposure, whereas the transcription rates of the two other genes *CaH αN* and *β* did not change after either kind of metal exposure, compared to their respective control levels in the midgut gland (Figure 5). In the mantle, however, the transcription levels of all three *Hc* genes remained unaffected in Cd‐ and Cu‐treated snails, when compared to their respective control levels (Figure 6).

### 3.4 Mapping of RNAseq data against *hemocyanin* and *MT* reference gene sequences

Trimmed reads of RNA sequences from midgut gland of control and Cu‐exposed snails (*n* = 2 each) were mapped against reference sequences of *CaH* genes *αD*, *αN*, and *β*, and the two *MT* genes *CdMT* and *CuMT*, to assess their relative quantity within the respective transcriptomes. As seen in Table 3, the transcriptomic mapping data seem to confirm the results derived from mRNA quantification by qRT‐PCR (Figures 3 and 6), showing a clear trend of higher transcript abundances for the gene *CaH αD* and the *CuMT* gene in the midgut gland of Cu‐exposed snails versus controls. In spite of the fact that these results must be considered with caution because of the small sample size (*n* = 2 of each treatment group), they seem to confirm the much more robust real‐time PCR data.

**Table 3:** *Hc* and *MT* gene expression in controls and Cu‐treated snails

	Co1	Co2	Cu1	Cu2
αD	3.6	2.9	11.1	19.0
αN	31.3	31.7	25.7	54.4
β	38.5	47.7	32.8	64.4
CuMT	38.4	12.9	176.9	174.3
CdMT	15.5	21.9	48.9	23.8

*Note*: Gene expression patterns in midgut gland of control (Co1, Co2) and Cu‐exposed snails (Cu1, Cu2) as inferred from transcriptomic mappings against reference sequences for *MT* (*CdMT* and *CuMT*) and Hc genes (*αD*, *αN*, *β*), expressed as gene‐specific transcripts per million.

## 4 DISCUSSION

### 4.1 Organ‐specific metal accumulation: Cd detoxification versus Cu regulation

Although in the present study only the midgut gland and the mantle of *C. aspersum* were considered, the observed patterns of Cd and Cu accumulation in these organs (see Figure 1 and Table 2) are consistent with previous findings in terrestrial helicid snails. Whereas Cd accumulates nearly exclusively in the midgut gland (Dallinger & Wieser, 1984; Dallinger et al., 1989), Cu is distributed more evenly among several snail tissues (Dallinger, 1993; Hispard et al., 2008). Elevated levels of this metal are normally found in the midgut gland, the mantle, the foot, and the kidney, especially after Cu exposure (Berger & Dallinger, 1989; Boshoff et al., 2013; Nowakowska et al., 2012). Apart from metal‐ and tissue‐specific accumulation patterns, an important difference between Cd and Cu exists with respect to their kinetic and metabolic behavior. Although at low environmental Cd concentrations some helicid snail species are able to excrete the metal through excretion via their mucus and feces (Notten et al., 2006), higher Cd levels in the substrate lead to a strong accumulation of the metal in the midgut gland due to a high efficiency of metal absorption versus low excretion rates (Berger & Dallinger, 1989). This eventually results in a progressive increase of metal levels and a persistent Cd storage in this organ on a long‐term scale (Dallinger & Wieser, 1984; Williamson, 1980). In contrast to Cd, Cu accumulation in snail organs occurs at lower levels and in a fluctuating manner, increasing during phases of Cu exposure, and decreasing down to basal levels after discontinuation of exposure (Dallinger & Wieser, 1984). These two different behavior patterns have been explained by storage detoxification for the nonessential metal Cd, and by physiological regulation for the essential trace element Cu (Dallinger, 1993; Dallinger et al., 2005). Many studies have shown that low external Cd concentrations can have a significant impact on internal tissue concentrations of essential metals like Zn or Cu, including plants (Borowska et al., 2017; Webster et al., 2011), vertebrates (Aydin et al., 2001; Kamunde & MacPhail, 2011; Pelgrom et al., 1995) and even snails (Nica et al., 2019). Although Nica et al. (2019) found an increase of internal Cu concentrations in the midgut gland of Cd‐exposed *C. aspersum*, this effect could not be observed in the present study, probably due to the higher metal concentrations applied in our exposure experiments. Notably at moderately elevated and high concentrations, the pathways of Cd and Cu in helicid snails are largely controlled by two metal‐selective MT isoforms, one detoxifying Cd in the midgut gland (called CdMT), the second one being associated with Cu (called CuMT) which is exclusively expressed in rhogocyte cells (Chabicovsky et al., 2003; Dallinger et al., 1997). As mentioned above, these cells can be scattered through connective tissues of all main snail organs (Haszprunar, 1996; Stewart et al., 2014). Consequently, the metabolic pathways of Cd and Cu in snail tissues remain strictly separated (Dallinger, 1996). This ensures that the molecular interactions between the two metals in snail cells and tissues can be minimized, a circumstance that might be important in view of the crucial role attributed to Cu in connection with Hc synthesis and metabolism (Dallinger et al., 2005).

### 4.2 Transcriptional upregulation of Cd‐ and Cu‐specific *MT* genes

The two metal‐specific *MT* genes (*CdMT* and *CuMT*) of *C. aspersum* exhibit tissue‐specific basal transcription patterns with high expression levels of *CdMT* in the midgut gland and clearly elevated basal transcription rates of *CuMT* in the mantle (Figure 2). This is consistent with the perception that the expressed CdMT protein in the midgut gland plays an important role in Cd detoxification, whereas the elevated *CuMT* expression in the mantle may reflect the presence of the CuMT isoform in the highly abundant rhogocytes within this tissue (Chabicovsky et al., 2003; Sairi et al., 2015).

In accordance with the predicted metal‐selective roles of the expressed CdMT and CuMT proteins (Höckner et al., 2011; Palacios et al., 2011), our results revealed also a metal and organ‐specific transcriptional upregulation of the *CdMT* and *CuMT* genes in *C. aspersum* (Figure 3). Whereas *CdMT* gene expression was manifold upregulated in the midgut gland and mantle of Cd‐exposed individuals, *CuMT* transcription was significantly upregulated only in the midgut gland of Cu‐stressed snails. Cd‐dependent *CdMT* gene upregulation was so far observed in all studies using terrestrial snail species of the clade of Stylommatophora (Palacios et al., 2011; Pedrini‐Martha et al., 2020), including *C. aspersum* (Höckner et al., 2011). In contrast, only transient or no upregulation at all of snail *CuMT* genes can be detected after Cu exposure (Höckner et al., 2011; Palacios et al., 2011). This suggests that *CuMT* gene upregulation in snails may occur at low and not always detectable rates, depending perhaps on its transient variability and on sample‐specific peculiarities such as the abundance of rhogocytes in the respective tissue preparations. On the other hand, our results confirm that the CuMT in snail rhogocytes may predominantly serve homeostatic regulation of intracellular Cu levels (Dallinger et al., 2005) with only transient upregulation peaks during phases of intracellular Cu excess. Rhogocytes are one of the most important cell types for Cu metabolism in gastropods for several reasons. First, they are the main sites of CuMT expression (Chabicovsky et al., 2003; Dallinger et al., 2005). Secondly, excessive amounts of Cu are detoxified in rhogocytes by storage compartmentalization into so‐called granules which are quickly formed upon excessive Cu exposure (Dallinger et al., 2005). Thirdly, rhogocytes are the main sites for synthesis of the gastropod respiratory proteins, namely hemoglobin or Hc, as demonstrated for *Haliotis tuberculata* (Albrecht et al., 2001), *Megathura crenulata* (Martin et al., 2011), *Lymnaea stagnalis* (Kokkinopoulou et al., 2015), or *Biomphalaria glabrata* (Kokkinopoulou et al., 2014). It was, therefore, hypothesized that the CuMT in rhogocytes may act as a donor or acceptor of Cu^+^ ions during Hc synthesis or degradation (Dallinger et al., 2005). Alternatively, it can be speculated that CuMT may function as a Cu transporter involved in the generation of rhogocyte Cu‐granules.

### 4.3 Tissue‐specific *hemocyanin* gene expression and its response to metal stress

Of the three *Hc* genes (*αD*, *αN*, and *β*) identified in *C. aspersum*, *CaH β* exhibits the highest basal transcription levels in both, midgut gland and mantle tissues (Figure 4). Overall, the expression of all three *Hc* genes appears to be higher in the mantle edge compared to the midgut gland. This is perhaps because the Hc demand of the mantle tissue may be particularly high, considering its involvement in oxygen uptake in pulmonate land snails. Cell and tissue‐specific as well as developmental expression patterns of Hc isoform genes have been observed in other gastropod species too, including *Haliotis asinina* (Streit et al., 2005) and *H. laevigata* (Sairi et al., 2015). Multiplication of *Hc* genes with differential expression patterns may have fostered the adaptation capacity of gastropods to novel habitats during evolution, extending their repertoire to respond to a variety of physiological and environmental conditions (Schäfer et al., 2019). Also, in nongastropod molluscs such as cephalopods, multiple *Hc* genes are expressed that probably possess different oxygen affinities under varying environmental conditions (Melzner et al., 2007).

Cd exposure, especially over a long‐term range, can induce an increased oxygen demand in exposed molluscs such as marine snails (Dalla Via et al., 1989) and oysters (Lannig et al., 2008). A possible response strategy to compensate for this higher oxygen demand would be an increased expression of the oxygen‐carrying protein Hc. In the present study, however, the transcription of Hc genes in Cd‐exposed *C. aspersum* was not upregulated at all. This may be the case in other mollusc species, too, which makes them particularly vulnerable to increased temperatures owing to climatic changes, especially in combination with environmental metal pollution (Lannig et al., 2008).

All the more so surprising was the observation that one of the *Hc* genes, specifically *CaH αD*, was upregulated in the midgut gland of Cu‐exposed snails (Figure 6 and Table 3). Since rhogocytes have been shown to be the only cells that synthesize Hc in the midgut gland of gastropods (Albrecht et al., 2001; Martin et al., 2011; Streit et al., 2005), it is highly probable that upregulation of *CaH αD* occurs predominantly in rhogocytes. Apart from Cu regulation and Hc synthesis (Dallinger et al., 2005), these cells seem to also possess their own metal detoxification mechanisms which rely on intracellular compartmentalization of toxic metals within granular vesicles (Dallinger et al., 2005; Kokkinopoulou et al., 2015; Simkiss & Mason, 1983), rather than on metal complexation by CdMT, that seems to prevail in snail tissues outside of rhogocytes (Chabicovsky et al., 2003). Therefore, compartmentalization of toxic metals (including Cd) within granular rhogocyte vesicles represents an alternative detoxification pathway when Cd‐specific MTs are not present. Hence, we assume that mollusc rhogocytes are, in addition to their specific functions in Hc synthesis, highly capable of metal stress resistance (Kokkinopoulou et al., 2014, 2015). This ability may be crucial to ensure nonimpairment of Cu pathways during Hc synthesis. Hence, the observed upregulation of the Hc isoform *CaH αD* in the midgut gland of Cu‐treated snails may reflect a role of the isoform in stress resistance, rather than indicating a direct connection between an increased Cu supply and Hc synthesis. In fact, intraspecific multiplicity of Hc isoforms may translate into functional specification of single isoforms, including their involvement in stress resistance like innate immunity, as well as antiviral or antibacterial activity (Wu et al., 2016; Yao et al., 2019). In the cuttlefish *Sepia officinalis*, several *Hc* genes are expressed differentially depending on the developmental stage and on adaptation to external stressors such as temperature of hypercapnia (Strobel et al., 2012). Also, in *Sepiella maindroni* Hc was upregulated after hypoxia or bacterial challenge (Li et al., 2017).

### 4.4 CuMT expression and Hc metabolism: A direct connection?

In a previous study with the Roman snail, *Helix pomatia*, it has been shown that the essential trace element Cu is strictly regulated in rhogocytes by diverting excess amounts of the metal into an intracellular granular pool, whereas the level of the Cu associated with the expressed CuMT protein remained fairly stable (Dallinger et al., 2005). The selective Cu binding by CuMT, its Cu‐buffering function, and its coexpression with a respiratory Cu‐protein in snail rhogocytes gave rise to the hypothesis that in these animals CuMT may be involved in Hc synthesis (Dallinger et al., 1997), serving perhaps as a Cu donator to nascent Hc molecules (Dallinger et al., 2000; Dallinger et al., 2005). Such a hypothesis is supported by findings that in arthropods and particularly in crustaceans, too, Cu‐specific MT isoforms can deliver Cu to arthropod Hcs (Brouwer et al., 1986; Brouwer et al., 2002), which in turn may also be involved in moulting and metal stress resistance (Engel & Brouwer, 1987; Engel et al., 2001).

The simultaneous upregulation of *CuMT* and Hc *CaH αD* genes in the midgut gland of Cu‐exposed snails as shown in the present study (Figures 3 and 6) is not a convincing argument to support the hypothesis of a Cu donation process through presumed interactions between CuMT and Hc molecules. Rather, it is assumed that the transcriptional upregulation of both genes might be a response to a stressful situation induced by Cu exposure.

## 5 CONCLUSIONS

The tissue specific accumulation pattern of Cd and Cu as well as the metal‐selective upregulation of the respective *Cd* or *CuMT* genes in *C. aspersum* reflects separated metabolic and detoxification pathways of these two metals within helicid snails. Whereas Cd is detoxified by binding to the CdMT protein, excessive amounts of Cu are scavenged in Cu granule within rhogocytes. In this study, gene transcription of all three *Hc* genes (*CaH αD*, *CaH αN*, *CaH β*) in *C. aspersum* was quantified for the first time, showing tissue and isoform specific expression patterns. Interestingly, *CaH αD* gene expression is moderately upregulated due to Cu‐exposure. However, a possible role in Cu detoxification still has to be elucidated.

## CONFLICT OF INTERESTS

The authors declare that there are no conflict of interests.

## Data Availability

The raw data for this study can be provided upon request by the corresponding authors through the server of the University of Innsbruck.
